# Heart rate variability in middle-aged adults: Use of Sasang typology to distinguish individuals susceptible to stress

**DOI:** 10.1097/MD.0000000000017764

**Published:** 2019-11-01

**Authors:** Hyunjoo Oh, Seungwon Shin, Byung-Hee Koh, Minwoo Hwang

**Affiliations:** a Department of Clinical Korean Medicine, Graduate School, Kyung Hee University; b Medical & Oriental Comprehensive Healthcare Center, Kyung Hee University Hospital; c Clinical Trial Center, Korean Medicine Hospital; d Department of Sasang Constitutional Medicine, College of Korean Medicine, Kyung Hee University, 26 Kyungheedae-ro, Dongdaemun-gu, Seoul, Republic of Korea.

**Keywords:** heart rate variability, Sasang constitutional medicine, So-Eum, stress, susceptibility to stress, traditional Korean medicine

## Abstract

While stress is known to cause many diseases, there is no established method to determine individuals vulnerable to stress. Sasang typology categorizes humans into four Sasang types (So-Eum, Tae-Eum, So-Yang, and Tae-Yang), which have unique pathophysiologies because of their differential susceptibilities to specific stimuli, including stress. The purpose of this study was to determine if Sasang typology can be used identify individuals who are vulnerable to stress by evaluation of heart rate variability (HRV).

This was a cross-sectional study. A total of 399 healthy men and women aged 30 to 49 years were recruited. Physical examinations for stress included HRV measurement and blood tests. The subjects also completed questionnaires about psychological stress, self-awareness, and lifestyle. HRV was analyzed using frequency-domain analysis. Subjects were divided into So-Eum (SE) and non-So-Eum (non-SE) groups according to their diagnosis.

The weight and body mass index in the SE group were significantly lower than those in the non-SE group (both, *P* *=* .000). There were no significant between-group differences in any other demographic variables. In HRV analysis, the normalized high frequency (nHF) was higher (*P* = .008) while the normalized low frequency (nLF; *P* = .008) and LF:HF ratio (LF/HF; *P* = .002) were lower in the SE group than in the non-SE group.

Although there was no difference in variables affecting HRV, HRV values were significantly different between groups. The LF/HF value for the SE group was at the lower limit of the normal range, although there were no associated clinical problems. These findings suggest that individuals with the SE type are more susceptible to stress than those with the other types. Thus, middle-aged individuals who are vulnerable to stress can be identified using Sasang typology.

## 1 Introduction

Stress is known to cause many disorders and complicate existing conditions, particularly in patients with cardiovascular disease, depression, and HIV/AIDS.^[1]^ Exogenous stress induces neuroendocrine and neurohumoral changes, immune alterations, autonomic and cardiovascular dysregulation, central neurotransmitter system dysfunction, and behavioral changes.^[2]^ However, not everyone who experiences stress develops consequent illnesses; individuals susceptible to stress and stress-related illnesses and the timing of onset of these conditions remain unclear. The concept of diathesis has been developed to account for these individual differences.

The term diathesis is synonymous with vulnerability, which is considered inherent within an individual and typically conceptualized as being stable over the life span. Some individuals are more vulnerable than others to stress-induced disease. According to the diathesis-stress model, a disorder is the result of an interaction between a predisposition to vulnerability and stress caused by daily life experiences.^[3]^ Although it is easy to explain that an individual is vulnerable to stress, an individual with diathesis cannot be identified on this basis. Fifty years later, attempts have been made to elucidate the diathesis using a person's genotype,^[4]^ although the results remain unclear. One new analytical tool that could complement this is Sasang typology.

Sasang typology, also known as Sasang constitutional medicine (SCM), is a branch of personalized medicine in Traditional Korean Medicine (TKM) that categorizes humans into 4 Sasang types using their psychological, physical, and genetic characteristics. The 4 Sasang types are So-Eum (SE), Tae-Eum (TE), So-Yang (SY), and Tae-Yang (TY).^[5]^ Each type has a unique pathophysiology because of their differential susceptibilities to specific stimuli, including stress and various disease outbreaks.^[6]^ Previous studies have revealed that the specific Sasang type could be considered a risk factor for certain noninflammatory chronic diseases, such as prehypertension, diabetes mellitus, general obesity, abdominal obesity, and metabolic syndrome.^[6–11]^

The present study investigated whether Sasang typology can be used to distinguish individuals who are vulnerable to stress by evaluation of heart rate variability (HRV). HRV refers to changes in the heart rate that are regulated by antagonism of the autonomic nervous system. Specific fluctuations in the time between successive heart beats provide information about autonomic nervous system function.^[12]^ HRV is a useful test for noninvasive evaluation of autonomic nervous system function.^[13]^ When the human body is threatened, continuous perception of that threat induces stress responses in the body. Threat perception and representation are governed by the amygdala, which is inhibited by the ventromedial prefrontal cortex (vmPFC).^[14,15]^ These inhibitory prefrontal processes can be assessed by HRV measurement. HRV can measure the degree of functional integration of the axes connecting vmPFC, the brainstem, and the peripheral anatomy and demonstrate how the autonomic nervous system is flexibly controlled. If the inhibitory process mentioned above is not functioning adequately, excessive stress responses will develop in the body, resulting in abnormal low frequency (LF)/high frequency (HF) values.^[16]^ According to a previous study, HRV can be affected by nonmodifiable determinants (age, sex, and race), physiological determinants (posture, systolic blood pressure, and heart rate), and lifestyle factors (mental stress, smoking, and alcohol consumption).^[17]^

In SCM, the SE type is characterized by a negative and self-directed personality and a nervous mind, which affects the unique symptomatology of this type.^[18]^ Previous studies have reported that individuals with the SE type are vulnerable to diseases related to the gastrointestinal system, such as irritable bowel syndrome^[19]^ and functional dyspepsia.^[20]^ Moreover, it has been hypothesized that the relationship between the SE type and a disease is mediated by the response to psychological stress.^[19]^ When stress occurs, processes to ensure allostasis occur in the body; allostasis refers to the dynamic equilibrium between several systems according to the brain output, which involves autonomic nervous system regulation.^[21]^ It can be assumed that individuals with the SE type exhibit poor allostatic regulation of the autonomic nervous system, relative to those with the other types. The hypothesis for the present research was that HRV would be unhealthier in individuals with the SE type than in those with the other Sasang types, because the formers are considered to be more sensitive to stress in SCM.

## 2 Methods

This study followed the Standard Protocol Items: Recommendations for Interventional Trials (SPIRIT) 2013 statement.^[22]^ The study protocol and informed consent guidelines were reviewed and approved by the Institutional Review Board of the Kyung Hee University Korean Medicine Hospital at Gangdong, Republic of Korea in September 2016 (KHNMCOH 2016-07-004-001) and October 2017 (KHNMCOH 2017-08-005-002). The protocol was registered with the Clinical Research Information Service (https://cris.nih.go.kr/cris/en/) on August 4, 2017 (KCT0002403) and October 13, 2017 (KCT0002768). The data were collected at the Korean Medicine Data Center of the Korea Institute of Oriental Medicine between September 2016 and March 2018.

### 2.1 Study design and subjects

**Figure 1:**
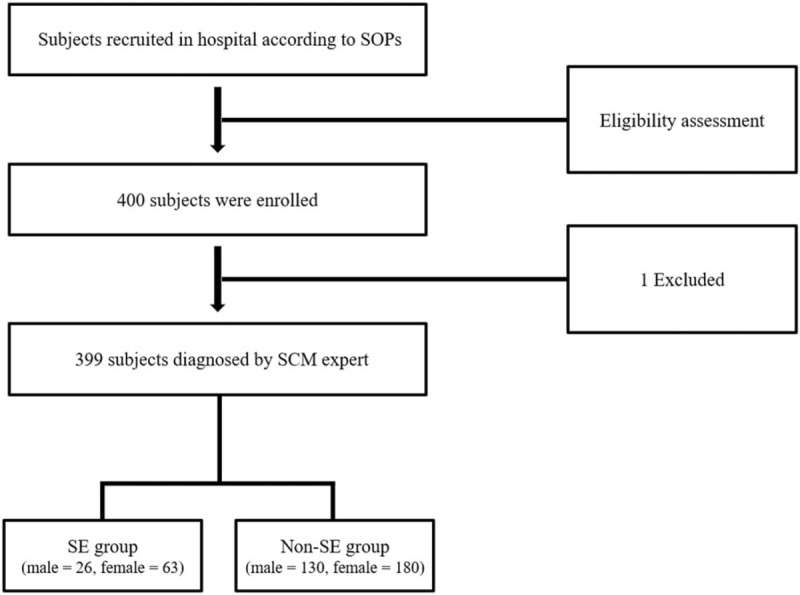
Flowchart of participant enrollment. SCM = Sasang constitutional medicine, SE = So-Eum type, SOP = standard operating procedure.

This was a cross-sectional study. In total, 400 healthy adults were enrolled from the Kyung Hee University Korean Medicine Hospital at Gangdong, Seoul. All subjects received an explanation of the study and provided informed consent. Physical examination, including HRV evaluation and blood tests, were performed for all subjects, who were also required to complete self-reported questionnaires regarding psychological stress, self-awareness, and lifestyle. Subjects fasted for at least 8 hours prior to the visit. Consequently, they were categorized into 2 groups: SE group, including those diagnosed with the SE type; and non-SE group, including those diagnosed with another type. Data for a total of 399 subjects were analyzed; 1 subject with missing data was excluded (Fig. 1).

### 2.2 Eligibility assessment

Healthy men and women aged 30 to 49 years old were included. Subjects were considered healthy when they exhibited a normal state in health checkups within the last 2 years and met 2 or less of the diagnosis criteria for metabolic syndrome in the screening test conducted at the visit. The criteria for metabolic syndrome followed those of the National Cholesterol Education Program – Third Adult Treatment Panel guidelines: fasting blood glucose ≥ 100 mg/dL or use of antihyperglycemic medication, triglycerides (TG) ≥150 mg/dL, serum high-density lipoprotein cholesterol ≤40 mg/dL for men and 50 mg/dL for women; systolic blood pressure ≥130 mm Hg and/or diastolic blood pressure ≥85 mm Hg or use of antihypertensive medication, and central obesity with a waist cutoff value of ≥90 cm for men and ≥85 cm for women.^[23]^ For abdominal obesity, we used a modified waist cutoff value for men and women.^[24]^ Participants with clinically significant medical conditions in their medical history/concomitant medication reviews and physical examinations were excluded. Participants who were involved in other trials within the previous month or pregnant at baseline were also excluded.

### 2.3 Data collection

#### 2.3.1 HRV analysis

To control for diurnal variation, HRV was measured between 9 and 12 am using the SA-3000P model (Medicore Co., Gyeonggi-do, Korea). The measurement laboratory was soundproof, lit by a fluorescent lamp, and maintained at room temperature. All subjects were instructed to avoid drinking alcohol and caffeinated beverages, smoking, and eating for least 8 hours before the HRV measurements.

All subjects were asked to remove metal accessories before the test, open their eyes, and adopt a comfortable sitting position in the chair. To control for biases introduced by posture changes or movement, subjects were asked not to move and to speak and breathe naturally during the test. To measure HRV, electrode sensors were placed on the inside of both wrists and the left ankle for 5 minutes. Subsequently, 1 sensor was additionally attached to their left index finger for 1.5 minutes.

A frequency-domain analysis method was selected because frequency-based analyses provide more discrete parsing of the frequency components of HRV and, consequently, clearer interpretation of parasympathetic and sympathetic influences than do time-domain analyses.^[25]^ We adopted the widely used spectral analysis, which partitions the total variance in HRV into low-frequency (LF: 0.04–0.15 Hz) and high-frequency (HF: 0.15–0.40 Hz) spectral power/variance bands.^[26]^ The analysis parameters are as follows: total power (TP), normalized LF (nLF), normalized HF (nHF), and LF:HF ratio (LF/HF). nLF, nHF, and LF/HF are considered to provide information about sympathovagal balance or sympathetic modulation.^[27]^

#### 2.3.2 Questionnaires

The Psychosocial Well-Being Index-Short Form (PWI-SF) was used to assess the degree of subjective mental stress. The PWI-SF is based on Goldberg General Health Questionnaire^[28]^ and comprises 18 items that are scored on a 4-point Likert scale. Scores from 0 to 3 were used to indicate the following responses, respectively: “always,” “frequently,” “sometimes,” and “not at all.” Negative items were reversed. A higher total score indicates higher subjective stress and poorer psychosocial health. Lifestyle activities, including alcohol consumption and smoking, were also evaluated.

#### 2.3.3 Physical examination

During the visit, the height, weight, and body mass index (BMI) of all participants were measured using the stadiometer BSM370 and Inbody 770 (Inbody CO., Seoul, Korea). Blood pressure was measured using an automatic digital blood pressure monitor (FT-500R PLUS, SELVAS Healthcare Inc., Seoul, Korea). Height and weight were measured in increments of 0.1 cm and 0.1 kg, respectively. Blood pressure was measured from the left upper arm at rest. Blood samples were obtained by peripheral venous puncture, and levels of total cholesterol (TC), triglyceride (TG), low-density lipoprotein cholesterol (LDL), high-density lipoprotein cholesterol (HDL), and hemoglobin A1c (HbA1c) were measured.

#### 2.3.4 Diagnosis of Sasang typology

The experts that diagnosed the Sasang typology had obtained professional qualifications from national accredited SCM programs and had more than 10 years of clinical experience. Diagnoses were based on the subjects’ appearance, which was assessed using a 3-dimensional facial scanner (Morpheus 3D NEO, Morpheus Cor., Gyeonggi-do, Korea), somatic morphology, and symptoms. Changes over time were also taken into account.

### 2.4 Statistical analysis

Student *t* tests were used to compare mean differences in continuous variables between groups, after testing for normality using the Kolmogorov–Smirnov test and Levene test. Between-group differences in categorical variables were tested using chi-square tests. All statistical analyses were performed using SPSS software version 18 (IBM, NY). A *P*-value of <.05 was considered statistically significant.

## 3 Results

### 3.1 Demographic data

Descriptive statistics for the SE and non-SE groups are provided in Table 1. There were no significant between-group differences in age, social habits such as drinking and smoking, and subjective stress levels. There was no difference in height, although both weight and BMI were significantly lower in the SE group than in the non-SE group (*P* = .000 and .000). Blood pressure, the mean heart rate, and blood lipid and HbA1c levels were also significantly lower in the SE group; however, all values were within the normal clinical limits.

**Table 1:**
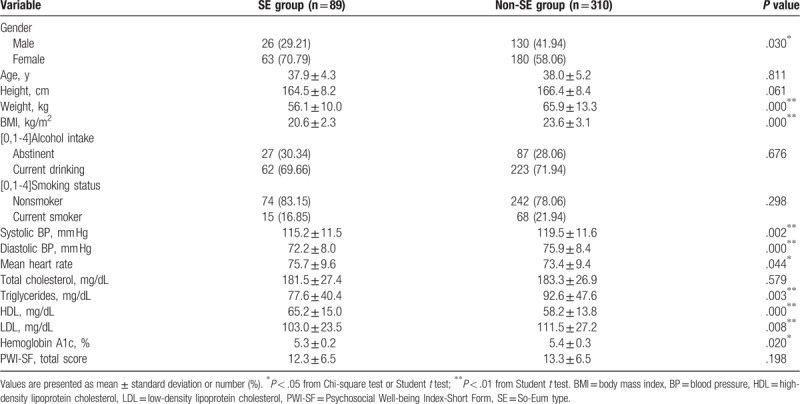
Demographic data of all subjects by group.

### 3.2 HRV results

HRV differences for the SE and non-SE groups are shown in Table 2. There was no significant between-group difference in TP. LF/HF and nLF were significantly lower (*P* = .008 and .002) while nHF was significantly higher (*P* = .008) in the SE group than in the non-SE group. All HRV values were within previously defined normal ranges,^[29]^ although the LF/HF value for the SE group was at the lower limit of this range.

**Table 2:**
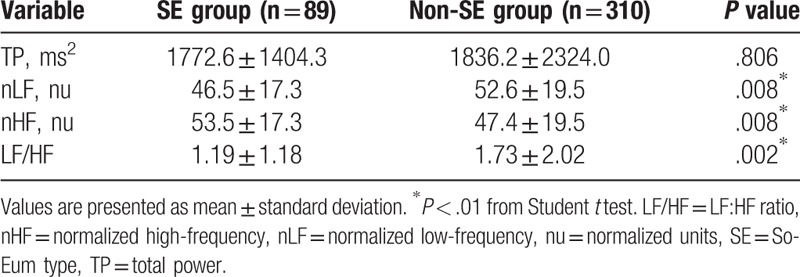
Heart rate variability in each group.

## 4 Discussion

In this study, we investigated whether SCM can be used to identify individuals vulnerable to stress via HRV measurements. All subjects were middle-aged and within the normal range for good health, and there were no significant differences in age, posture, and lifestyle. This also included the subjective stress level, which was not high according to the score-based classification in a previous study.^[19]^ There was no between-group difference in the intensity of stress judged by the subjects themselves in the questionnaire. However, the LF/HF value was at the lower limit of normal in the SE group, even though there were no associated clinical problems. This suggests that the autonomic nervous system of these patients was not balanced and out of the normal range. Therefore, it can be assumed that the inhibitory prefrontal processes applied to the amygdala are insufficient in the SE type. These results also indicate individual differences in the degree to which psychological stress manifests clinically in individuals with the different Sasang types. Further studies are necessary. Previous research has evaluated the relationship between obesity and autonomic nervous system imbalances.^[30]^ However, no study has assessed autonomic nervous system imbalances within the normal BMI range. In the present study, the BMI value for the SE group was within the normal range, although it was lower than that for the non-SE group. However, the LF/HF value for the SE group was not in the physiologically healthy range. This suggests that the use of Sasang typology is beneficial for screening individuals with weak regulation of autonomic nervous system functions in a normal BMI group.

In SCM, the SE type is characterized by weak digestion due to its inherent sensitive and anxious nature, resulting in a smaller body size and lower BMI relative to those in individuals with the other Sasang types.^[31]^ In other words, a lower BMI in individuals with the SE type is caused by constitutional characteristics and used an indicator for diagnosis of the Sasang type. Therefore, it was not considered an independent factor for HRV in this study. Subsequent studies correcting BMI between Sasang groups are necessary. In addition, it is necessary to confirm whether the potential stress on the body actually increases the incidence of disease by observing health changes and the development of diseases in individuals with the SE type and those with the other types.

This study has several limitations. First, it was restricted to middle-aged individuals. The subjects were not randomly selected from the general population, and the sex ratio was not corrected. In addition, all variables were measured only once, leading to the possibility of measurement errors, particularly in HRV. Meanwhile, the accuracy or consistency of SCM diagnoses remains unclear. However, this study excluded the possibility that HRV values are affected by clinical problems, because all subjects were healthy. In conclusion, the findings of this study suggest that middle-aged individuals who are vulnerable to stress can be distinguished using Sasang typology. Moreover, there is a relationship between vulnerability to stress and physiological function or pathological outcomes in individuals with the SE type. Further studies with participants from a wider age range and prospective periods of observation are necessary.

## Acknowledgment

The authors thank the Bio & Medical Technology Development Program of the National Research Foundation funded by the Ministry of Science and ICT (NRF-2014M3A9D7034367 & NRF-2014M3A9D7045482) and the Traditional Korean Medicine Research & Development Program funded by the Ministry of Health & Welfare through the Korea Health Industry Development Institute (HB16C0055) for the support. The funder played no role in the study design, data collection, study management, and data analysis or interpretation.

## Author contributions

**Conceptualization:** Byung-Hee Koh, Minwoo Hwang.

**Investigation:** Hyunjoo Oh.

**Methodology:** Seungwon Shin.

**Project administration:** Byung-Hee Koh, Minwoo Hwang.

**Visualization:** Hyunjoo Oh, Seungwon Shin.

**Writing – original draft:** Hyunjoo Oh.

**Writing – review & editing:** Seungwon Shin, Byung-Hee Koh, Minwoo Hwang.
